# Preliminary evaluation of the protective effects of recombinant AMA1 and IMP1 against *Eimeria stiedae* infection in rabbits

**DOI:** 10.1186/s13071-022-05492-4

**Published:** 2022-10-31

**Authors:** Jie Xiao, Ruoyu Zheng, Xin Bai, Jiayan Pu, Hao Chen, Xiaobin Gu, Yue Xie, Ran He, Jing Xu, Bo Jing, Xuerong Peng, Guangyou Yang

**Affiliations:** 1 Department of Parasitology, College of Veterinary Medicine, Sichuan Agricultural University, Wenjiang, 611130 China; 2 Department of Chemistry, College of Life and Basic Science, Sichuan Agricultural University, Wenjiang, 611130 China

**Keywords:** *Eimeria stiedae*, AMA1, IMP1, Recombinant protein-based subunit vaccine, Protective efficacy

## Abstract

**Background:**

*Eimeria stiedae* parasitizes the bile duct, causing hepatic coccidiosis in rabbits. Coccidiosis control using anticoccidials led to drug resistance and residues; therefore, vaccines are required as an alternative control strategy. Apical membrane antigen 1 (AMA1) and immune mapped protein 1 (IMP1) are surface-located proteins that might contribute to host cell invasion, having potential as candidate vaccine antigens.

**Methods:**

Herein, we cloned and expressed the *E. stiedae Es*AMA1 and *Es*IMP1 genes. The reactogenicity of recombinant AMA1 (r*Es*AMA1) and IMP1 (r*Es*IMP1) proteins were investigated using immunoblotting. For the vaccination-infection trial, rabbits were vaccinated with r*Es*AMA1 and r*Es*IMP1 (both 100 μg/rabbit) twice at 2-week intervals. After vaccination, various serum cytokines were measured. The protective effects of r*Es*AMA1 and r*Es*IMP1 against *E. stiedae* infection were assessed using several indicators. Sera were collected weekly to detect the specific antibody levels.

**Results:**

Both r*Es*AMA1 and r*Es*IMP1 showed strong reactogenicity. Rabbits vaccinated with r*Es*AMA1 and r*Es*IMP1 displayed significantly increased serum IL-2 (*F*_(4, 25)_ = 9.53, *P* = 0.000), IL-4 (*F*_(4, 25)_ = 7.81, *P* = 0.000), IL-17 (*F*_(4, 25)_ = 8.55, *P* = 0.000), and IFN-γ (*F*_(4, 25)_ = 6.89, *P* = 0.001) levels; in the r*Es*IMP1 group, serum TGF-β1 level was also elevated (*F*_(4, 25)_ = 3.01, *P* = 0.037). After vaccination, the specific antibody levels increased and were maintained at a high level. The vaccination-infection trial showed that compared with the positive control groups, rabbits vaccinated with the recombinant proteins showed significantly reduced oocyst output (*F*_(5, 54)_ = 187.87, *P* = 0.000), liver index (*F*_(5, 54)_ = 37.52, *P* = 0.000), and feed conversion ratio; body weight gain was significantly improved (*F*_(5, 54)_ = 28.82, *P* = 0.000).

**Conclusions:**

r*Es*AMA1 and r*Es*IMP1 could induce cellular and humoral immunity, protecting against *E. stiedae* infection. Thus, r*Es*AMA1 and r*Es*IMP1 are potential vaccine candidates against *E. stiedae*.

**Graphic abstract:**

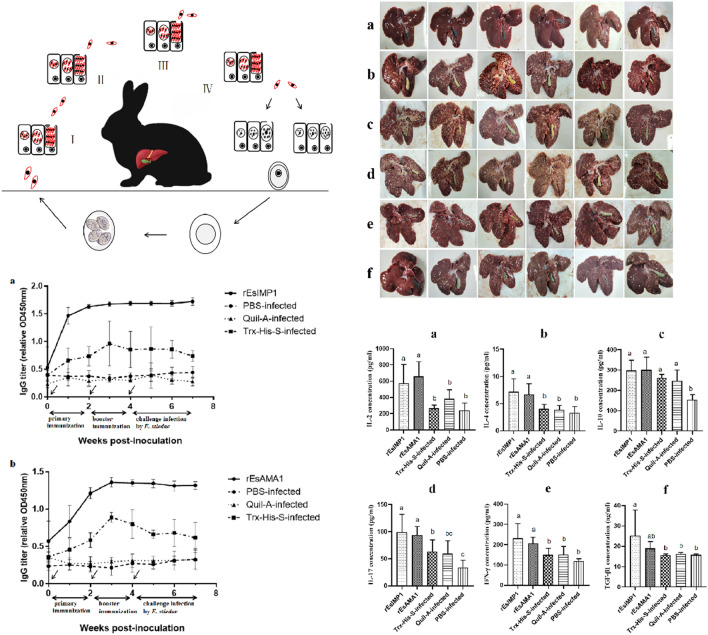

## Background

Rabbit coccidiosis is a highly contagious protozoan disease, which can affect rabbits of all ages, especially those between 1 and 4 months old [1]. Coccidiosis in adult rabbits is usually subclinical and asymptomatic. However, adults can become carriers and suffer from poor feed conversion and growth performance [2, 3]. Among the *Eimeria* species that infect rabbits, only *Eimeria stiedae* parasitizes the liver bile duct [4, 5]. *Eimeria stiedae* completes its endogenous stages in rabbit bile duct epithelial cells and causes liver dysfunction during its reproduction, resulting in severe hepatic coccidiosis [6].

Currently, rabbit coccidiosis control relies mainly on anticoccidials, which have led to problems such as drug resistance and residues [7]. Vaccines are promising alternative control strategies for chemoprophylaxis. However, live vaccines are relatively expensive and carry the risk of pathogen transmission or reversal of virulence [8]. Recombinant subunit vaccines may circumvent these drawbacks [8]. Coccidia undergo four life-cycle stages and need to migrate in the host; therefore, their antigenic composition is very complex, making screening of protective antigens for next-generation vaccines particularly important [9]. Studies have screened for candidate antigens [10]; however, only CoxAbic^®^ (Netanya, Israel) is currently commercialized [11].

Apical membrane antigen 1 (AMA1) is a key molecule for apicomplexans to invade host cells [12]. Recently, research on AMA1 as an antigen has shown a certain level of protection against apicomplexan infections such as *Plasmodium* spp. [13], *Toxoplasma gondii* [14], *Babesia* spp. [15], and *Eimeria* spp. [16]. Immune mapped protein 1 (IMP1), identified in 2011, is a surface-located protein that might contribute to host cell invasion [17]. In subsequent research, recombinant *Emax*IMP1 induced protection against *E. maxima* infection [18]. Furthermore, the homologous gene of IMP1 was found in *Toxoplasma gondii* and *Neospora caninum* [19, 20]. So far, there has been no report of a recombinant subunit vaccine for rabbit coccidia. In this study, the *EsAMA1* and *EsIMP1* genes were selected based on *E. stiedae* transcriptome data for prokaryotic expression [21]. Then, the recombinant proteins, r*Es*AMA1 and r*Es*IMP1, were used as subunit vaccines. The results showed that r*Es*AMA1 and r*Es*IMP1 conferred immune protection against *E. stiedae* by stimulating both humoral and cellular immune responses. This research provides a reference for developing a recombinant protein-based subunit vaccine for *E. stiedae*.

## Methods

### Parasites, Animals, and Sera

The *E. stiedae* Sichuan strain was propagated in our laboratory. Sixty coccidia-free New Zealand White rabbits (45 days old, 1.086 ± 0.068 kg, 30 females and 30 males), with five female and five male rabbits in each group, were randomly grouped. Experimental groups included the r*Es*IMP1 and r*Es*AMA1 groups (r*Es*IMP1 or r*Es*AMA1 proteins vaccinated and *E. stiedae* infected). Positive control groups included PBS-infected (sterile phosphate-buffered saline mock-vaccinated and *E. stiedae* infected), Quil-A-infected (saponin derivative Quil-A mock vaccinated and *E. stiedae* infected), and Trx-His-S-infected (pET-32a tag protein mock vaccinated and *E. stiedae* infected) groups. The PBS-uninfected group comprised sterile phosphate-buffered saline mock vaccination without *E. stiedae* infection (Table 1). The rabbits were housed in pairs in flame-sterilized steel cages, with a plastic partition placed at the bottom to prevent contact with feces. The rabbits were raised based on the method described by Wei’s research [22]. Anticoccidial drugs were discontinued 1 week before the challenge infection was performed, and pathogenic examination was performed every other day to ensure that no coccidia oocysts were detected. The rabbits were vaccinated with a bivalent vaccine against rabbit hemorrhagic disease virus and *Pasteurella multocida* at 35 days old.

**Table 1:** Trial design and vaccine procedures

Groups	Number of rabbits	Immunogen and dosage	Vaccination weeks	Vaccination route	infection dose/week/route
PBS-uninfected	10	1 ml Sterile PBS	0, 2	Subcutaneous injection in the neck	–
PBS-infected	10	1 ml Sterile PBS	0, 2	Subcutaneous injection in the neck	1 × 10^4^ sporulated oocysts/week 4/oral
Quil-A-infected	10	1 mg Quil-A dilution in 1 ml PBS	0, 2	Subcutaneous injection in the neck	1 × 10^4^ sporulated oocysts/week 4/oral
Trx-His-S-infected	10	100 μg Trx-His-S tag + 1 mg Quil-A dilution in 1 ml PBS	0, 2	Subcutaneous injection in the neck	1 × 10^4^ sporulated oocysts/week 4/oral
r*Es*IMP1	10	100 μg r*Es*IMP1 + 1 mg Quil-A dilution in 1 ml PBS	0, 2	Subcutaneous injection in the neck	1 × 10^4^ sporulated oocysts/week 4/oral
r*Es*AMA1	10	100 μg r*Es*AMA1 + 1 mg Quil-A dilution in 1 ml PBS	0, 2	Subcutaneous injection in the neck	1 × 10^4^ sporulated oocysts/week 4/oral

Rabbits were inoculated orally with 5 × 10^4^ *E. stiedae* sporulated oocysts for positive sera collection. Negative sera were obtained from the 1-month-old coccidia-free rabbits without past exposure to any *Eimeria* species. All sera were stored at − 20 °C.

### Bioinformatic analysis

The open reading frames (ORF) and amino acid sequences of *EsAMA1* and *EsIMP1* were obtained using ORF Finder (https://www.ncbi.nlm.nih.gov/orffinder/). The ExPASy Proteomics Server (http://web.Expasy.org/protparam/) was used to predict the molecular weight (MW) of the proteins. The TMHMM Server v.2.0 (http://www.cbs.dtu.dk/services/TMHMM/#opennewwindow) and the SignalP4.1 (http://www.cbs.dtu.dk/services/SignalP/) server were used to analyze the transmembrane regions and signal peptides of the proteins, respectively. B-cell epitopes were predicted using the IEBD Analysis Resource (http://tools.immuneepitope.org/bcell/). The multiple sequence alignment was performed using Jalview 2.11.2.0 [23].

### Cloning, expression, and protein purification

Total RNA of *E. stiedae* sporulated oocysts was extracted using a commercial kit (Tiangen, China). First-strand cDNA was synthesized from the total RNA and then used for second-strand cDNA synthesis (Thermo, Waltham, MA, USA). The *EsAMA1* and *EsIMP1* specific primers were designed based on transcriptome data [21]: *Es*AMA1-F 5′-CGGGATCCATGTGGAAGATGAGGCTTGT-3′, EsAMA1-R 5′-CCCTCGAGTTAAAAGTCCTGGTCAACGAG -3′, with *BamH*I and *Xho*I restriction enzyme sites (underlined) (Takara, Dalian, China); *Es*IMP1-F 5′-CGGGATCCATGGGGGCCCTCTGTTCG-3′, *Es*IMP1-R 5′-GCGTCGACTCAATCATCTTGCTTCTCCTGCTG-3′, with *BamH*I and *Sal*I restriction enzyme sites (underlined).

The *EsAMA1* and *EsIMP1* genes were amplified by polymerase chain reaction (PCR), and the amplicons were sequenced (Sangon, Shanghai, China). The target *EsAMA1* and *EsIMP1* fragments were digested with *BamH*I/*Xho*I and *BamH*I/*Sal*I, respectively, and then ligated into the expression vector pET32a( +) (Takara). Then, *Escherichia coli* BL21 (DE3) was used to express the proteins (induced by 1 mM isopropyl β-d-1-thiogalactopyranoside). r*Es*AMA1 and r*Es*IMP1 were purified using a Nuvia Ni-Charged IMAC Cartridge (Bio-Rad, Hercules, CA, USA). After purification, r*Es*IMP1 and r*Es*AMA1 were analyzed using sodium dodecyl sulfate polyacrylamide gel electrophoresis (SDS-PAGE). The purified fusion Trx-His-S tag protein (with no insert fragment) was cryopreserved in our laboratory. The recombinant protein that was expressed in the inclusion bodies of *E. coli* was dialyzed at 4 °C according to the method detailed in Shi’s study [24].

### Western blotting analysis

The separated r*Es*AMA1 and r*Es*IMP1 were transferred onto nitrocellulose (NC) membranes (Boster, Wuhan, China), separately. After blocking for 2 h using Tris-buffered saline (TBS) containing 5% (*w/v*) skimmed milk at room temperature, the NC membranes were incubated with positive or negative sera (1:200 *v/v* dilution) overnight at 4 °C. The membranes were washed and incubated with horseradish peroxidase (HRP)-conjugated secondary antibodies (EarthOx Life Sciences, Millbrae, CA, USA, 1:2000 *v/v* dilution) for 2 h at room temperature. Then, detection of the specific bands was performed using a Metal Enhanced DAB Substrate Kit (20 ×) (Solarbio, Beijing, China) after further washing.

### Design of the vaccination-infection trial

**Fig. 1:**
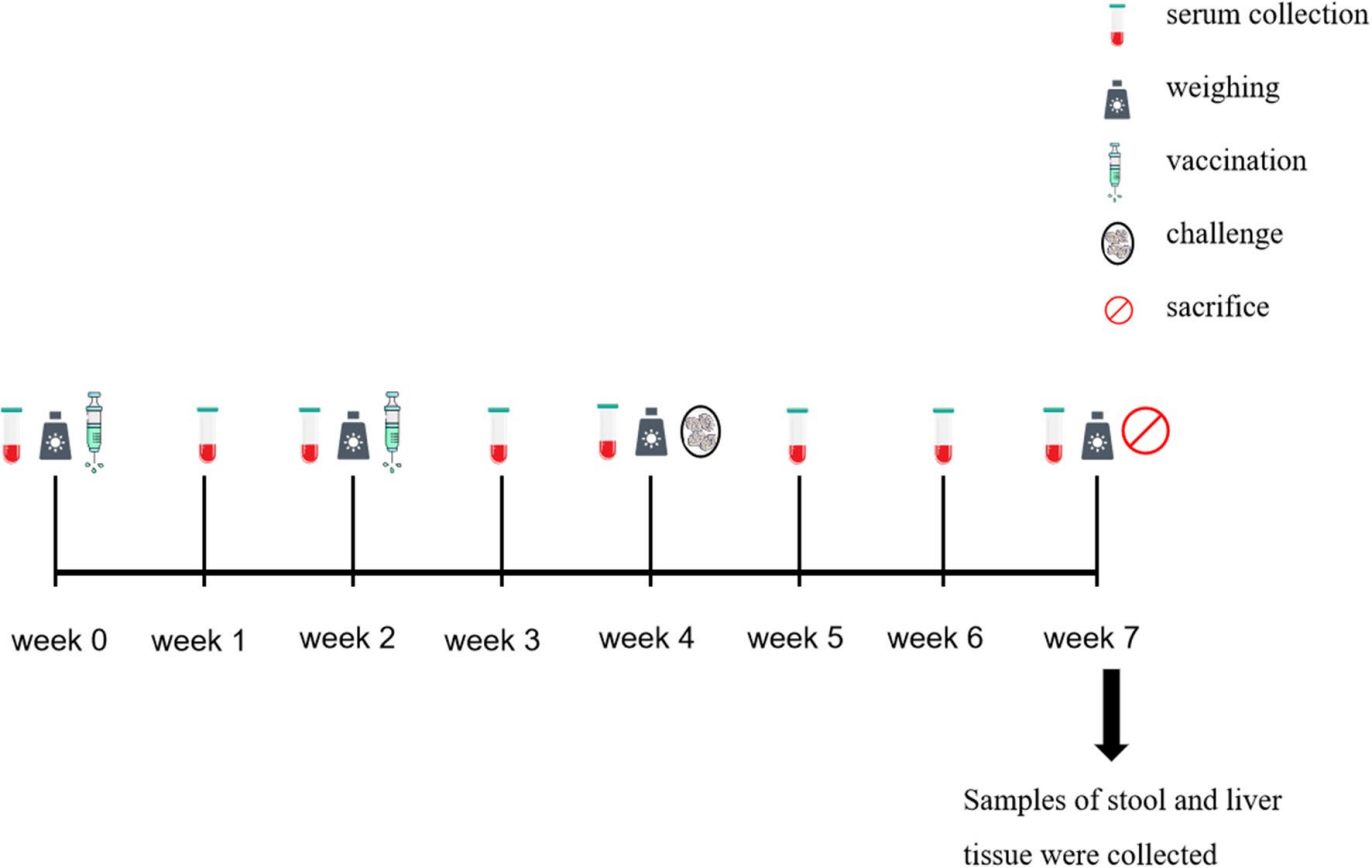
Time course of the collection of the samples

The details of trial design and vaccine procedures are summarized in Table 1. The timings of sample collection are shown in Fig. 1.

### Evaluation of protective effect

Safety evaluation: The health status of all experimental rabbits was observed after vaccination. The bodyweight of each rabbit was recorded before the first vaccination, booster vaccination, and infection. The weight gain after vaccination was determined as the weight before infection minus the weight before the first vaccination to verify whether the vaccination affects the weight gain of the experimental rabbits.

The protective effects of r*Es*AMA1 and r*Es*IMP1 against *E. stiedae* infection were assessed according to several indicators, calculated as follows: (1) The survival rate in each group was obtained by dividing the number of surviving rabbits by the initial number of rabbits. (2) The body weight gain after infection = the weight before sacrifice − the weight before infection. (3) After sacrifice, 2 g of feces was collected from the rectum, and the McMaster method was used to calculate the amount of oocysts excreted per gram of feces (OPG) [25]. (4) Liver index = (liver weight/weight before sacrifice) × 100%. (5) Feed conversion ratio = feed consumption (g)/the rabbits mass after infection (g).

### Estimation of serum AST/ALT levels

Blood samples were collected into vacuum blood collection tubes without any anticoagulants before sacrifice. The serum alanine aminotransferase (AST) and aspartate aminotransferase (ALT) levels were then measured using enzyme-linked immunosorbent assay (ELISA) kits (RUIXIN Biotech, Quanzhou, China).

### Determination of serum anti-rEsAMA1 and rEsIMP1 IgG levels

Pre-vaccine sera (week 0) were collected, and then sera were collected weekly after vaccination. All sera were stored at − 20 °C. Specific antibody levels in the sera (OD_450_ values of serum samples) were evaluated using indirect ELISAs based on the recombinant proteins r*Es*AMA1 and r*Es*IMP1. The concentrations of the recombinant proteins and sera were determined using standard checkerboard titration procedures [26]. The optimal concentration of r*Es*AMA1 was 0.78 μg/well, while it was 0.96 μg/well for r*Es*IMP1. The optimal serum dilution was 1:160.

### Detection of serum cytokine levels

The levels of circulating interleukin (IL)-2, IL-4, IL-10, IL-17, interferon gamma (IFN-γ), and transforming growth factor beta 1 (TGF-β1) were estimated after two vaccinations using ELISAs. The rabbit IFN-γ ELISA kit was purchased from MABTECH (Nacka Strand, Sweden), and the other ELISA kits were purchased from CUSABIO (Wuhan, China).

### Statistical analysis

The differences among the groups were assessed using one-way analysis of variance (ANOVA) employing IBM SPSS statistics 22.0 (IBM Corp., Armonk, NY, USA). *P* < 0.05 was considered significant, and *P* < 0.01 was considered extremely significant.

## Results

### Cloning and bioinformatic analysis

The sequences of *EsAMA1* (GenBank accession number: MZ934414) and *EsIMP1* (GenBank accession number: MZ934415) were successfully amplified. The ORF of *EsAMA1* was 1644 bp (encoding a protein with a predicted MW of 60 kDa). *Es*AMA1 has a predicted transmembrane region (amino acids 458–480), but no predicted signal peptide. The ORF of *EsIMP1* was 1191 bp (encoding a protein with a predicted MW of 43 kDa). *Es*IMP1 has no predicted signal peptide or transmembrane region.

**Fig. 2:**
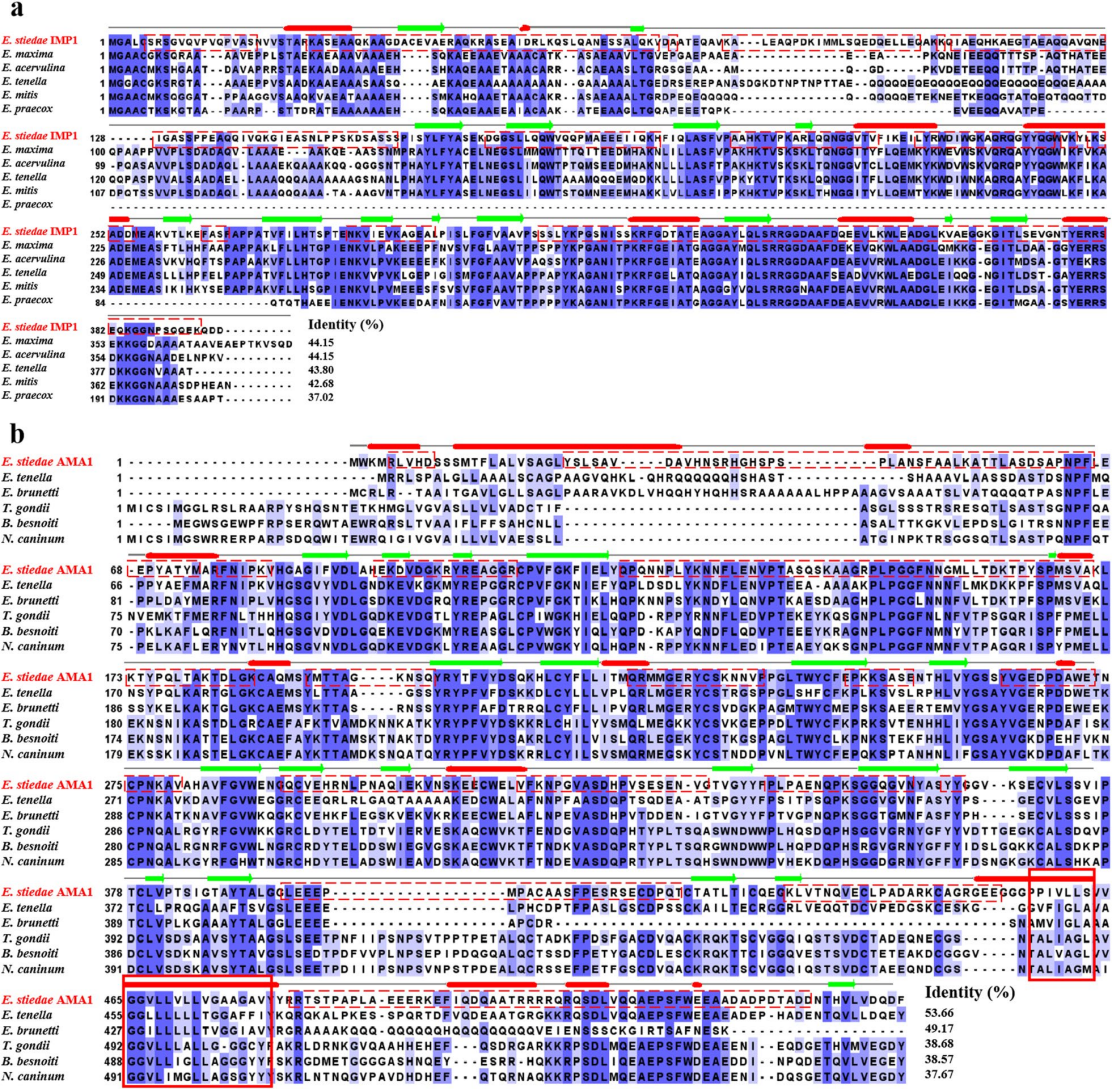
Multiple sequence alignments of IMP1 and AMA1 from different species. **a** Multiple alignment of *E. stiedae* IMP1 with IMP1 proteins from other parasites: *Eimeria maxima* (UniProt: F4MKA6), *Eimeria acervulina* (UniProt: U6GHD9), *Eimeria tenella* (UniProt: F4MKA7), *Eimeria mitis* (UniProt: U6JVY4), *Eimeria praecox* (UniProt: U6GBP2); (**b**) multiple alignment of *E. stiedae* AMA1 with AMA1 proteins from other parasites: *Eimeria tenella* (UniProt: U6KTA0), *Eimeria brunetti* (UniProt: U6LBB9), *Toxoplasma gondii* (UniProt: B6KAM0), *Besnoitia besnoiti* (UniProt: A0A2A9MBX4), *Neospora caninum* (UniProt: F0VH85); Blue shading indicates conserved residues. Dashed red boxes represent B-cell epitopes. The transmembrane region is marked with a solid red box

Multiple sequence alignments revealed that *Es*IMP1 and *Es*AMA1 proteins are highly variable (Fig. 2). The amino acid sequences of *Es*IMP1 and *Es*AMA1 shared 37.02–44.15% and 37.67–53.66% identity with IMP1 and AMA1 proteins from different Apicomplexan species, respectively.

### Expression, purification, and western blotting analysis of rEsAMA1 and rEsIMP1

**Fig. 3:**
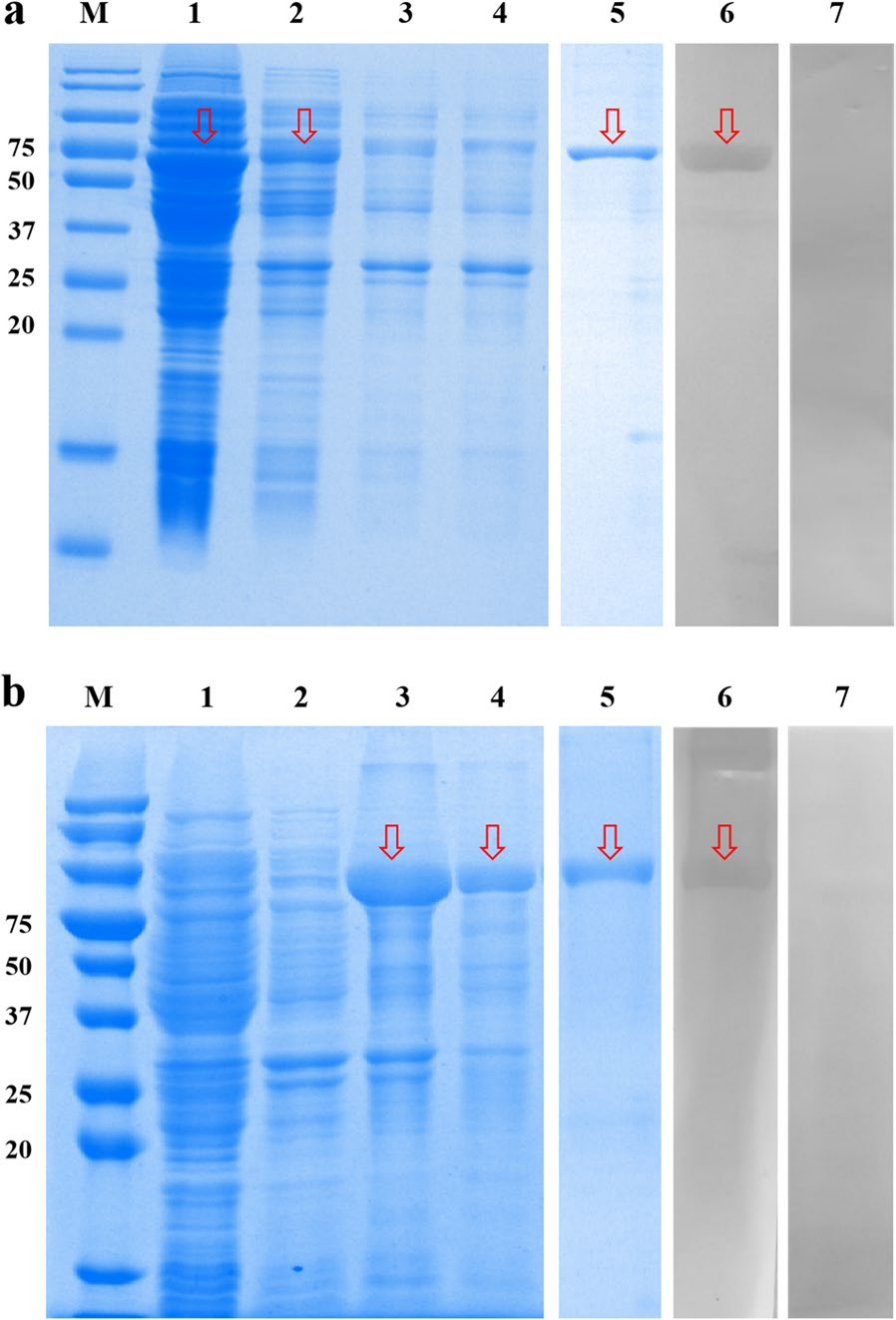
SDS-PAGE and Western blotting analysis of r*Es*IMP1 (**a**) and r*Es*AMA1 (**b**). Lane M: Protein molecular weight markers; lanes 1–4: recombinant proteins that were dissolved in the supernatant, 4 M urea, 6 M urea, and 8 M urea after ultrasonication; lane 5: purified recombinant proteins; lane 6: purified recombinant proteins incubated with anti-*E. stiedae* positive sera; lane 7: purified recombinant proteins incubated with negative sera from coccidia-free rabbits (the bands are indicated using arrows)

r*Es*IMP1 (~ 63 kDa) was expressed in the supernatant, and r*Es*AMA1 (~ 80 kDa) was expressed in inclusion bodies of *E. coli*. r*Es*AMA1 was mainly dissolved in 6 M and 8 M urea (Fig. 3, lanes 1–4). The MW of the recombinant proteins included the approximately 20-kDa fusion tag protein encoded by vector pET32a( +). After purification using an Ni^2+^ affinity column, r*Es*IMP1 and r*Es*AMA1 were analyzed using SDS-PAGE (Fig. 3, lane 5). Purified r*Es*AMA1 was dialyzed at 4 ℃ according to the method detailed in Shi’s study [26,24].

r*Es*IMP1 and r*Es*AMA1 reacted with anti-*E. stiedae* positive sera and specific bands were observed on the NC membranes (Fig. 3, lane 6), while incubation with the sera from coccidia-free rabbits showed no specific bands (Fig. 3, lane 7). These results indicated that both r*Es*IMP1 and r*Es*AMA1 have strong reactogenicity.

### Evaluation of the protective effect of rEsAMA1 and rEsIMP1

No statistically significant differences were observed for weight gain after vaccination among the six groups (*F*_(5, 54)_ = 0.16, *P* = 0.977) (Table 2). No obvious adverse reactions were observed in the vaccinated rabbits. This result suggested that the recombinant proteins r*Es*AMA1 and r*Es*IMP1 have good safety at the doses used in our experiments.

**Table 2:** Protective effects of r*Es*AMA1 and r*Es*IMP1 against *E. stiedae* infection under different evaluation indicators

Groups	Average body weight gain after vaccination (g)	Average body weight gain after infection (g)	Relative body weight gain rate (%)	Oocyst shedding per rabbit (× 10^5^/g)	Oocyst decrease ratio (%)	Average liver index	Feed conversion ratio	Survival rate (%)
PBS-uninfected	902.00 ± 90.16^a^	656.60 ± 99.5^a^	100	0^a^	–	3.06 ± 0.26^a^	3.20:1	100
PBS-infected	870.00 ± 42.69^a^	347.00 ± 104.25^b^	52.9	15.84 ± 2.42^b^	0	6.92 ± 0.94^b^	6.05:1	100
Quil-A-infected	907.00 ± 160.77^a^	361.80 ± 89.51^b^	55.2	16.57 ± 2.80^b^	− 4.6	6.84 ± 0.85^b^	5.80:1	100
Trx-His-S-infected	886.00 ± 105.75^a^	341.80 ± 78.95^b^	52.1	16.15 ± 2.12^b^	− 1.9	7.02 ± 1.21^b^	6.14:1	100
r*Es*IMP1	881.00 ± 105.67^a^	619.00 ± 70.00^a^	94.4	3.16 ± 0.66^c^	80.0	4.27 ± 0.68^c^	3.39:1	100
r*Es*AMA1	897.78 ± 116.06^a^	512.00 ± 48.26^c^	78.1	4.02 ± 0.39^c^	74.6	5.21 ± 0.88^d^	4.10:1	100

The data are presented as the mean ± standard deviation (SD). In each column, there is a significant difference between the data marked with different letters (a, b, c; ANOVA, *P* < 0.05), and there is no significant difference between the data marked with the same letter (*P* > 0.05)

**Fig. 4:**
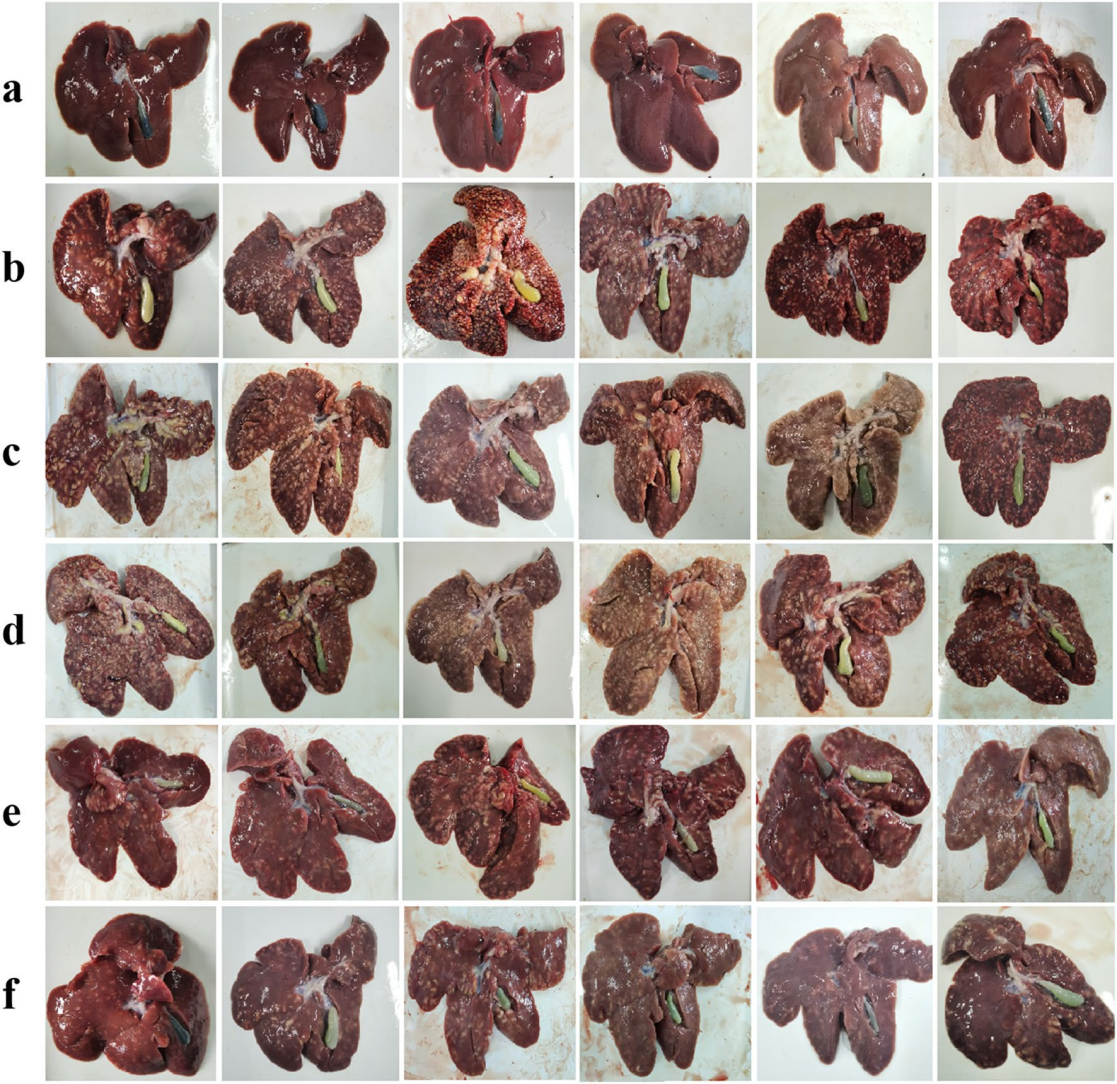
Gross postmortem examinations of the liver. **a** PBS-uninfected group; (**b**) PBS-infected group; (**c**) Quil-A-infected group; (**d**) Trx-His-S-infected group; (**e**) r*Es*AMA1 group; (**f**) r*Es*IMP1 group

Gross postmortem examination showed that the livers of the infected groups were enlarged, and their surfaces were full of different-sized and -shaped yellowish-white nodules. The gallbladders were distended with yellowish fluid (Fig. 4).

According to the survival rate, weight gain, oocyst output, liver index, and the feed conversion ratio, r*Es*AMA1 and r*Es*IMP1 showed good protection against *E. stiedae* infection (Table 2). The average body weight gain after infection in the two protein vaccinated groups (78.1% and 94.4%, respectively) was significantly higher than that in the three positive control groups: PBS-infected, Quil-A-infected, and Trx-His-S-infected groups (*F*_(5, 54)_ = 28.82, *P* = 0.000).

The oocyst output decreased significantly in the r*Es*AMA1 and r*Es*IMP1 groups (*F*_(5, 54)_ = 187.87, *P* = 0.000), displaying 74.6% and 80.0% oocyst output reductions, respectively.

The r*Es*AMA1 and r*Es*IMP1 groups had better feed conversion ratios compared with the positive control groups from week 4 to week 7. The feed conversion ratios of the r*Es*AMA1 and r*Es*IMP1 groups were 4.10:1 and 3.39:1, respectively, while the PBS-infected, Quil-A-infected, and Trx-His-S-infected groups reached ratios of 6.05:1, 5.80:1, and 6.14:1, respectively. The liver indices of the r*Es*AMA1 and r*Es*IMP1 groups were lower than those of the three positive control groups (*F*_(5, 54)_ = 37.52, *P* = 0.000).

### Estimation of serum AST/ALT levels in different groups of the experiment

Compared with the PBS-uninfected group, the serum ALT levels of the five infected groups increased significantly (*F*_(5, 30)_ = 3.43, *P* = 0.014). There was no statistical difference in serum AST levels (*F*_(5, 30)_ = 1.05, *P* = 0.408) (Table 3).

**Table 3:** Biochemical estimation of ALT and AST levels in the six groups

Groups	ALT (U/l)	AST (U/l)
PBS-uninfected	21.82 ± 1.86^a^	15.93 ± 1.55^a^
PBS-infected	30.33 ± 3.82^b^	19.34 ± 1.61^a^
Quil-A-infected	29.55 ± 3.15^b^	18.43 ± 3.58^a^
Trx-His-S-infected	29.05 ± 5.44^b^	18.74 ± 3.77^a^
r*Es*AMA1	27.96 ± 3.90^b^	18.23 ± 3.33^a^
r*Es*IMP1	28.75 ± 5.24^b^	17.45 ± 2.44^a^

The data are presented as mean ± standard deviation (SD). In each column, there is a significant difference between the data marked with different letters (a, b, c; ANOVA, *P* < 0.05), and there is no significant difference between the data marked with the same letter (*P* > 0.05)

### Serum anti-rEsAMA1 and rEsIMP1 antibody levels

**Fig. 5:**
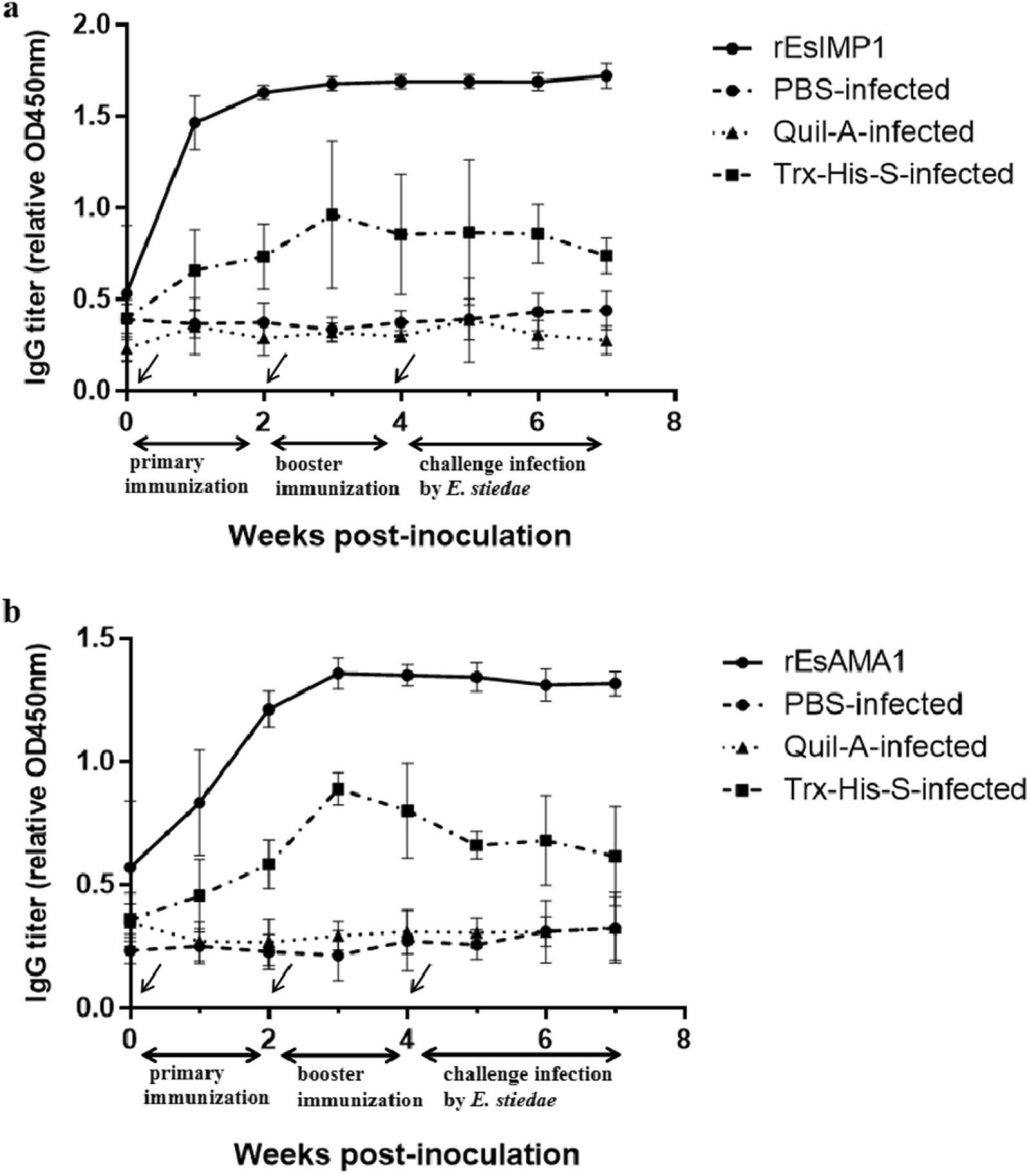
Changes in serum antibody levels. The changes in serum anti-r*Es*IMP1 (**a**) and r*Es*AMA1 (**b**) antibody levels after the first vaccination (week 0, indicated by arrows), booster vaccination (week 2, indicated by arrows), and infection with *E. stiedae* (week 4, indicated by an arrow)

In the r*Es*IMP1 group (Fig. 5a), the anti-r*Es*IMP1 antibody level increased rapidly after the first vaccination and reached its highest level in the second week. In the r*Es*AMA1 group (Fig. 5b), the anti-r*Es*AMA1 antibody level increased more slowly than that in the r*Es*IMP1 group and reached its highest level in the third week. After two vaccinations with r*Es*IMP1 and r*Es*AMA1, the specific antibody levels were maintained at a high level. In addition, the Trx-His-S-infected group also showed an increase in antibody levels, indicating that the inclusion of the Trx-His-S tag in the recombinant r*Es*IMP1 and r*Es*AMA1 proteins increased the antibody levels; however, the Trx-His-S-infected group’s antibody levels were lower than those of the r*Es*IMP1 and r*Es*AMA1 groups. The PBS-infected and Quil-A-infected groups showed almost no changes in antibody levels.

### Cytokine levels induced by rEsAMA1 and rEsIMP1

**Fig. 6:**
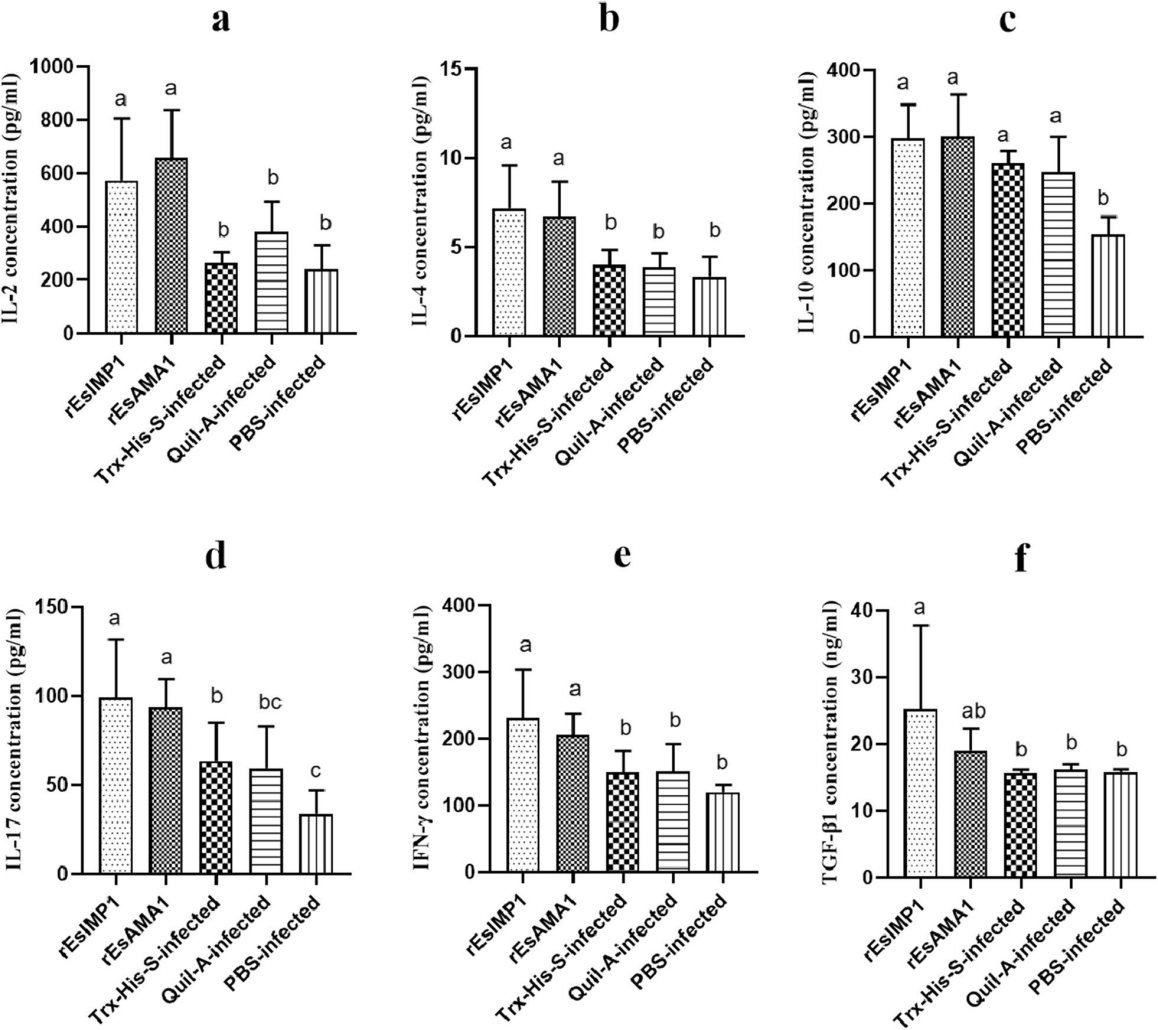
Post-vaccination cytokine levels. The serum IL-2 (**a**), IL-4 (**b**), IL-10 (**c**), IL-17 (**d**), IFN-γ (**e**), and TGF-β1 (**f**) levels at 2 weeks after booster vaccination. Different superscripts (**a**, **b**) indicate a significant difference (*P* < 0.05). The same superscript indicates no significant difference (*P* > 0.05). The unit of TGF-β1 concentration was ng/ml, and the concentration unit of other cytokines was pg/ml

The serum cytokine levels were estimated 2 weeks after the booster vaccination. Rabbits vaccinated with r*Es*AMA1 and r*Es*IMP1 displayed significantly increased serum levels of IL-2 (*F*_(4, 25)_ = 9.53, *P* = 0.000), IL-4 (*F*_(4, 25)_ = 7.81, *P* = 0.000), IL-17 (*F*_(4, 25)_ = 8.55, *P* = 0.000), and IFN-γ (*F*_(4, 25)_ = 6.89, *P* = 0.001); the TGF-β1 level was also elevated in the r*Es*IMP1 group (*F*_(4, 25)_ = 3.01, *P* = 0.037). The IL-10 level increased significantly in all groups except the PBS-infected group (*F*_(4, 25)_ = 10.53, *P* = 0.000) (Fig. 6).

## Discussion

At present, control of coccidiosis mainly relies on the addition of anticoccidials. However, the emergence of drug resistance and drug residues forced researchers to focus on vaccine development [7]. The precocious lines (PL) of *Eimeria intestinalis* [27], *Eimeria magna* [28], *Eimeria flavescens* [29], *Eimeria media* [30], and *Eimeria piriformis* [31] were selected in rabbits. The PL of *E. intestinalis* had strong immunogenicity: the vaccination of six oocysts was sufficient to reduce the oocyst output by about 60%, while vaccination with 600 or more oocysts provided complete protection in rabbits [32]. Mohamed et al. [33] reported a 97% oocyst output reduction in rabbits following vaccination with 3500 PL of *E. magna*. However, the PL of *E. flavescens* showed weak immunogenicity [34]. Additionally, it is possible to use the gamma-ray radiation-attenuated *Eimeria* spp. as a vaccine to prevent coccidiosis [35].

However, live anticoccidial vaccines have drawbacks such as high production cost and the risk of virulence reversal [8]. Therefore, it is necessary to explore a new generation of anticoccidial vaccines to overcome these shortcomings. Recombinant subunit vaccines are easier to mass produce than live vaccines and have a longer shelf life, making them an ideal alternative strategy [36]. In recent years, some antigens have shown good protective effects in chicken coccidiosis and have been identified as vaccine candidate antigens, including adhesion and invasion-related antigens [16, 18], sexual reproduction stage-related antigens [37], and common antigens [38]. Omata [39] and Hanada et al. [40] found that the soluble antigens in the bile from *E. stiedae*-infected rabbits could induce protection against *E. stiedae* infection. Rabbits vaccinated with *E. stiedae* coproantigen in Freund's adjuvant showed a high level of IgG response, and vaccination resulted in a decline in the oocyst count [41]. These studies showed that it is feasible to develop vaccines using the immunodominant antigens of rabbit coccidia.

AMA1 is involved in apicomplexan host cell invasion and has been considered a candidate vaccine antigen [12, 16]. Lei et al. [42] found that BALB/c mice vaccinated with r*Po*AMA1 (recombinant AMA1 protein from *Plasmodium ovale*) showed high antibody titers. r*Po*AMA1 induced IFN-γ-secreting cells and increased the lymphocyte proliferation response. In chickens, AMA1 has also been studied as a candidate vaccine antigen against coccidial infection [43, 44]. A previous study reported a 25.37–33.33% oocyst output reduction and a 75.45–81.50% weight gain in chickens following vaccination with live bacteria expressing *Et*AMA1 and infection with 4 × 10^4^ *E. tenella* sporulated oocysts [16]. Another study reported a 77.4% oocyst output reduction in chickens following vaccination with recombinant *E. tenella* AMA1 protein and infection with 250 *E. tenella* sporulated oocysts [44]. In our study, rabbits vaccinated with r*Es*AMA1 showed improved weight gain, and the reduction rate of oocyst output in feces reached 74.6%. We consider the reasons for the difference between studies to be as follows: (1) The two previous studies [16, 44] were performed with different infectious doses and *E. tenella* isolates. (2) The *E. stiedae* used in this study parasitizes the rabbit liver, and there are marked differences from chicken coccidia in terms of biological characteristics such as host, parasitic site, and pathogenicity, which will also lead to differences in the results.

Recombinant IMP1 could provide immune protection against chicken coccidiosis [18, 45–47]. In this study, rabbits vaccinated with r*Es*IMP1 displayed a 94.4% body weight gain and an 80.0% oocyst output reduction. Our results are similar to Yin’s reports [45, 47], in which chickens vaccinated with r*Et*IMP1 or its fusion expression product, r*Et*IMP1-CD40L, showed significantly reduced lesion scores, reduced oocyst output (by 62.58–77.6%), and a relative weight gain rate of 70.86–86.03%. Kundu et al. [46] found that in chickens vaccinated with r*Et*IMP1, the cecal parasite genome numbers were reduced by 67–79%. Based on the above test results, the recombinant proteins r*Es*AMA1 and r*Es*IMP1 showed good protective effects against *E. stiedae* infection.

Cellular immunity performs an essential function in defending the host from coccidiosis, whereas the effect of humoral immunity is limited [48, 49]. Th1-type cytokines, such as IFN-γ and IL-2, partially regulate T-cell responses against *Eimeria* [50, 51]. IFN-γ enhances macrophage, natural killer (NK) cell, and cytotoxic T lymphocyte (CTL) functions to defend against *Eimeria* infection [52]. IL-2 might contribute to the proliferation of T cells involved in cytotoxic effector mechanisms [53]. Early studies found that chicken IFN-γ has an anti-coccidial effect and can inhibit the development of *E. tenella* sporozoites [54]. *Eimeria* resistance of chickens might be weakened because of reduced IFN-γ and IL-2 levels [55]. Vaccination with expression products containing IFN-γ or IL-2 further improved the anticoccidial index of chickens [56, 57]. Quil-A can stimulate both humoral and cellular responses and enhance Th1 and CTL responses [58]. Quil-A, which is ideal for vaccines against intracellular pathogens, has been studied in *Toxoplasma gondii* and *Neurospora caninum* with good results [58, 59]. Therefore, we chose Quil-A as the adjuvant for the recombinant proteins in this study. Compared with the control groups, serum IL-2 and IFN-γ levels were significantly elevated after vaccination with r*Es*IMP1 and r*Es*AMA1 (*P* < 0.05), indicating that r*Es*IMP1 and r*Es*AMA1 can stimulate Th1-type immune responses. Additionally, the ability to elicit Th1-type cytokine response might be further enhanced by Quil-A. Similarly, the serum IL-4, IL-17, and TGF-β1 levels were also significantly increased in the r*Es*IMP1 and r*Es*AMA1 groups. The Th2-type immune response is characterized by elevated levels of IL-4 and other cytokines. IL-4 plays a role in regulating B cells and inducing humoral immune reactions [60, 61]. Thus, r*Es*IMP1 and r*Es*AMA1 can also stimulate a Th2-type immune response.

Recent research has proven that antibodies play a role during *Eimeria* infection [62]. Lee et al. [63, 64] found that in chickens fed with hyperimmune IgY of egg yolk powder, passive immunity provided significant protection against *Eimeria* infection. CoxAbic^®^ comprises *Eimeria maxima* gametocyte antigens, and breeder hens vaccinated with CoxAbic^®^ produced large amounts of specific IgY maternal antibodies, which provided passive immunity for their offspring against *Eimeria* [11]. We found that rabbits vaccinated with r*Es*IMP1 and r*Es*AMA1 exhibited elevated serum levels of specific antibodies. The results indicated that both r*Es*IMP1 and r*Es*AMA1 induce significant humoral immunity; however, r*Es*IMP1 displayed a better performance.

*Eimeria stiedae* parasitizes the rabbit liver and completes its endogenous stages in bile duct epithelial cells [6]. However, it is not yet clear how *E. stiedae* migrate from the duodenum to the liver. Studies found sporozoites of *E. stiedae* in the mesenteric lymph nodes (MLN) and supposed that the sporozoites might be transported to the liver via the portal vein and lymphatic system [65, 66]. Owen et al. [67] observed *E. stiedae* sporozoites in the MLN, bone marrow, liver, and plasma and proposed that sporozoites steadily accumulate in the MLN. Suo et al. [68] proposed that the sporozoites are transported via the lymphatic vessels to the MLN and are then carried by lymph into the systemic circulation, after which the sporozoites continue their migration and finally enter the bile duct epithelial cells via the capillaries of intrahepatic bile ducts. AMA1 and IMP1 are expressed at high level in sporozoites and are located on the sporozoites surface [69, 70]. The anti-r*Et*AMA1 polyclonal antibodies had a potent inhibitory effect on *E. tenella* sporozoite invasion, decreasing it by approximately 70% [69]. Treatment of *Neospora caninum* tachyzoites with anti-r*Nc*IMP1 polyclonal antibodies reduced cell invasion by approximately 44% [20]. Therefore, we speculated that when *E. stiedae* sporozoites migrate in the blood circulation, the anti-r*Es*AMA1 or anti-r*Es*IMP1 antibodies might interact with them and inhibit cell invasion by the sporozoites. Meanwhile, the high IFN-γ and IL-2 levels in the vaccinated rabbits further inhibited the intracellular infection of *E. stiedae*. Together, these effects might eventually lead to significant differences in oocyst output and body weight gain. There is currently no specific standard for the evaluation of recombinant subunit vaccines against rabbit coccidiosis; therefore, the protective efficacy was assessed through the survival rate, clinical symptoms, oocyst output reductions, and body weight gain. In the present study, rabbits vaccinated with r*Es*AMA1 or r*Es*IMP1 displayed a significantly reduced oocyst output (*F*_(5, 54)_ = 187.87, *P* = 0.000) and increased body weight gain (*F*_(5, 54)_ = 28.82, *P* = 0.000). Thus, r*Es*AMA1 and r*Es*IMP1 are potential candidate vaccines against *E. stiedae*.

## Conclusions

In this study, we obtained recombinant r*Es*IMP1 and r*Es*AMA1 proteins. The vaccination-infection trial showed that both r*Es*IMP1 and r*Es*AMA1 conferred protective immunity against *E. stiedae* infection. r*Es*IMP1 and r*Es*AMA1 could stimulate specific antibodies and the production of various cytokines. Thus, r*Es*IMP1 and r*Es*AMA1 could induce significant cellular and humoral immunity and alleviated the body weight loss and oocyst output, making them potential candidate vaccines against *E. stiedae*. The results showed that r*Es*IMP1 performed better than r*Es*AMA1.

## Data Availability

The datasets supporting the conclusions of this article are included within the article.

## References

[1] Pakandl M, Hlásková L, Poplstein M, Chromá V, Vodicka T, Salát J (2008). Dependence of the immune response to coccidiosis on the age of rabbit suckling. Parasitol Res.

[2] Jing F, Yin G, Liu X, Xun S, Qin Y (2011). Large-scale survey of the prevalence of *Eimeria* infections in domestic rabbits in China. Parasitol Res.

[3] Yin G, Goraya MU, Huang J, Suo X, Huang Z, Liu X (2016). Survey of coccidial infection of rabbits in Sichuan Province. Southwest China Springerplus.

[4] Kvicerová J, Pakandl M, Hypsa V (2008). Phylogenetic relationships among *Eimeria* spp. (Apicomplexa, Eimeriidae) infecting rabbits: evolutionary significance of biological and morphological features. Parasitology.

[5] Pakandl M (2009). Coccidia of rabbit: a review. Folia Parasitol (Praha).

[6] Hanada S, Omata Y, Umemoto Y, Kobayashi Y, Furuoka H, Matsui T (2003). Relationship between liver disorders and protection against *Eimeria stiedai* infection in rabbits immunized with soluble antigens from the bile of infected rabbits. Vet Parasitol.

[7] Noack S, Chapman HD, Selzer PM (2019). Anticoccidial drugs of the livestock industry. Parasitol Res.

[8] Peek HW, Landman WJM (2011). Coccidiosis in poultry: anticoccidial products, vaccines and other prevention strategies. Vet Q.

[9] Song H, Dong R, Qiu B, Jing J, Zhu S, Liu C (2017). Potential vaccine targets against rabbit coccidiosis by immunoproteomic analysis. Korean J Parasitol.

[10] Venkatas J, Adeleke MA (2019). A review of *Eimeria* antigen identification for the development of novel anticoccidial vaccines. Parasitol Res.

[11] Wallach M, Smith NC, Petracca M, Miller CM, Eckert J, Braun R (1995). *Eimeria maxima* gametocyte antigens: potential use in a subunit maternal vaccine against coccidiosis in chickens. Vaccine.

[12] Lamarque M, Besteiro S, Papoin J, Roques M, Vulliez-Le Normand B, Morlon-Guyot J (2011). The RON2-AMA1 interaction is a critical step in moving junction-dependent invasion by apicomplexan parasites. PLoS Pathog.

[13] Srinivasan P, Ekanem E, Diouf A, Tonkin ML, Miura K, Boulanger MJ (2014). Immunization with a functional protein complex required for erythrocyte invasion protects against lethal malaria. Proc Natl Acad Sci.

[14] Zhang TE, Yin LT, Li RH, Wang HL, Meng XL, Yin GR (2015). Protective immunity induced by peptides of AMA1, RON2 and RON4 containing T-and B-cell epitopes via an intranasal route against toxoplasmosis in mice. Parasit Vectors.

[15] Hidalgo-Ruiz M, Mejia-López S, Pérez-Serrano RM, Zaldívar-Lelo de Larrea G, Ganzinelli S, Florin-Christensen M (2022). *Babesia bovis* AMA-1, MSA-2c and RAP-1 contain conserved B and T-cell epitopes, which generate neutralizing antibodies and a long-lasting Th1 immune response in vaccinated cattle. Vaccine.

[16] Li J, Wang F, Ma C, Huang Y, Wang D, Ma D (2018). Recombinant *lactococcus lactis* expressing *Eimeria tenella* AMA1 protein and its immunological effects against homologous challenge. Exp Parasitol.

[17] Blake DP, Billington KJ, Copestake SL, Oakes RD, Quail MA, Wan KL (2011). Genetic mapping identifies novel highly protective antigens for an apicomplexan parasite. PLoS Pathog.

[18] Jenkins MC, Stevens L, Brien CO, Parker C, Miska K, Konjufca V (2018). Incorporation of a recombinant *Eimeria maxima* IMP1 antigen into nanoparticles confers protective immunity against *E. **m**axima* challenge infection. Vaccine..

[19] Cui X, Lei T, Yang D, Hao P, Li B, Liu Q (2012). *Toxoplasma gondii* immune mapped protein-1 (TgIMP1) is a novel vaccine candidate against toxoplasmosis. Vaccine.

[20] Cui X, Lei T, Yang DY, Hao P, Liu Q (2012). Identification and characterization of a novel *Neospora caninum* immune mapped protein 1. Parasitology.

[21] Xie Y, Xiao J, Zhou X, Gu X, Yang G (2021). Global transcriptome landscape of the rabbit protozoan parasite *Eimeria stiedae*. Parasit Vectors.

[22] Wei W, Shen N, Xiao J, Tao Y, Yang G (2020). expression analysis and serodiagnostic potential of microneme proteins 1 and 3 in *Eimeria stiedai*. Genes (Basel).

[23] Waterhouse AM, Procter JB, Martin DMA (2009). Jalview Version 2 - a multiple sequence alignment editor and analysis workbench. Bioinformatics.

[24] Shi T, Zhang L, Li Z, Newton IP, Zhang Q (2015). Expression, purification and renaturation of truncated human integrin β1 from inclusion bodies of *Escherichia coli*. Protein Expr Purif.

[25] Bortoluzzi C, Paras KL, Applegate TJ, Verocai GG (2018). Comparison between mcmaster and mini-FLOTAC techniques for the enumeration of *Eimeria maxima* oocysts in poultry excreta. Vet Parasitol.

[26] Crowther JR. The ELISA guidebook. Methods in molecular biology (Clifton, NJ) Humana press New Jersey 2001 149. 10.1385/1592590497 11028258

[27] Licois D, Coudert P, Boivin M (1990). Selection and characterization of a precocious line of *Eimeria intestinalis*, an intestinal rabbit coccidium. Parasitol Res.

[28] Licois D, Coudert P, Drouet-viard F (1995). *Eimeria magna*: Pathogenicity, immunogenicity and selection of aprecociousline. Vet Parasitol.

[29] Pakandl M (2005). Selection of a precocious line of the rabbit coccidium *Eimeria flavescens* Marotel and Guilhon (1941) and characterisation of its endogenous cycle. Parasitol Res.

[30] Licois D, Coudert P, Drouet-viard F (1994). *Eimeria media*: Selection and characterization of a precocious line. Parasitol Res.

[31] Pakandl M, Jelínková A (2006). The rabbit coccidium *Eimeria piriformis*: Selection of a precocious line and life-cycle study. Vet Parasitol.

[32] Coudert P, Licois D, Provot F (1993). *Eimeria* sp. from the rabbit (*Oryctolagus cuniculus*): pathogenicity and immunogenicity of *Eimeria intestinalis*. Parasitol Res.

[33] Mohamed SB, Soraya T, Hassina A (2018). A vaccination trial with a precocious line of *Eimeria magna* in algerian local rabbits *Oryctolagus cuniculus*. Vet Parasitol.

[34] Pakandl M, Hlásková L, Poplstein M (2008). Immune response to rabbit coccidiosis: a comparison between infections with *Eimeria flavescens* and *E. intestinalis*. Folia Parasitol.

[35] Xiaoming Z (1986). The effects of gamma rays on the pathogenicity and immunogenicity of sporulated oocysts of Eimeria sliedai[J]. Journal Shandong Agric Univ.

[36] Shirley MW, Smith AL, Blake DP (2007). Challenges in the successful control of the avian coccidia. Vaccine.

[37] Belli SI, Mai K, Skene CD, Gleeson MT, Witcombe DM, Katrib M (2004). Characterisation of the antigenic and immunogenic properties of bacterially expressed, sexual stage antigens of the coccidian parasite *Eimeria maxima*. Vaccine.

[38] Liu T, Huang J, Ehsan M, Wang S, Hong F, Zhou Z (2018). Protective immunity against *Eimeria maxima* induced by vaccines of Em14-3-3 antigen. Vet Parasitol.

[39] Omata Y, Sueda M, Koyama T (2001). Identification and the role of soluble antigens detected in bile from *Eimeria stiedai*-infected rabbits. J Parasitol.

[40] Hanada S, Umemoto Y, Omata Y (2003). *Eimeria stiedai* merozoite 49-kDa soluble antigen induces protection against infection. J Parasitol.

[41] Abdel Megeed KN, Abuel Ezz NM, Abdel-Rahman EH (2005). Protective effect of *Eimeria stiedae* coproantigen against hepatic coccidiosis in rabbits. J Egypt Soc Parasitol.

[42] Lei Y, Shen F, Zhu H, Zhu L, Chu R, Tang J (2020). Low genetic diversity and strong immunogenicity within the apical membrane antigen-1 of plasmodium ovale spp. imported from Africa to China. Acta Trop.

[43] Liu Y, Jiang YL, Liu J, Gao X, Zhang Z, Huang HB (2020). Recombinant invasive *Lactobacillus plantarum* expressing the *Eimeria tenella* fusion gene TA4 and AMA1 induces protection against coccidiosis in chickens. Vet Parasitol.

[44] Pastor-Fernández I, Kim S, Billington K, Bumstead J, Marugán-Hernández V, Küster T (2018). Development of cross-protective *Eimeria*-vectored vaccines based on apical membrane antigens. Int J Parasitol.

[45] Yin G, Lin Q, Qiu J, Qin M, Tang X, Suo X (2015). Immunogenicity and protective efficacy of an *Eimeria* vaccine candidate based on *Eimeria tenella* immune mapped protein 1 and chicken CD40 ligand. Vet Parasitol.

[46] Kundu K, Garg R, Kumar S, Mandal M, Tomley FM, Blake DP (2017). Humoral and cytokine response elicited during immunisation with recombinant Immune mapped protein-1 (EtIMP-1) and oocysts of *Eimeria tenella*. Vet Parasitol.

[47] Yin G, Lin Q, Wei W, Qin M, Liu X, Suo X (2014). Protective immunity against *Eimeria tenella* infection in chickens induced by immunization with a recombinant C-terminal derivative of EtIMP1. Vet Immunol Immunopathol.

[48] Pierce AE, Long PL (1965). Studies on acquired immunity to coccidiosis in bursaless and thymectomized fowls. Immunology.

[49] Rose ME, Hesketh P (1979). Immunity to coccidiosis: T-lymphocyte- or B-lymphocyte-deficient animals. Infect Immun.

[50] Dalloul RA, Lillehoj HS (2005). Recent advances in immunomodulation and vaccination strategies against coccidiosis. Avian Dis.

[51] Abdel-Haleem HM, Aboelhadid SM, Sakran T, El-Shahawy G, El-Fayoumi H, Al-Quraishy S (2017). Gene expression, oxidative stress and apoptotic changes in rabbit ileum experimentally infected with *Eimeria intestinalis*. Folia Parasitol (Praha).

[52] Lillehoj HS (1998). Role of T lymphocytes and cytokines in coccidiosis. Int J Parasitol.

[53] Choi KD, Lillehoj HS (2000). Role of chicken IL-2 on gammadelta T-cells and *Eimeria acervulina*-induced changes in intestinal IL-2 mRNA expression and gammadelta T-cells. Vet Immunol Immunopathol.

[54] Lillehoj HS, Choi KD (1998). Recombinant chicken interferon-gamma-mediated inhibition of *Eimeria tenella* development in vitro and reduction of oocyst production and body weight loss following *Eimeria acervulina* challenge infection. Avian Dis.

[55] Isobe T, Lillehoj HS (1993). Dexamethasone suppresses T cell-mediated immunity and enhances disease susceptibility to *Eimeria mivati* infection. Vet Immunol Immunopathol.

[56] Song X, Huang X, Yan R, Xu L, Li X (2015). Efficacy of chimeric DNA vaccines encoding *Eimeria tenella* 5401 and chicken IFN-γ or IL-2 against coccidiosis in chickens. Exp Parasitol.

[57] Lillehoj HS, Choi KD, Jenkins MC, Vakharia VN, Song KD, Han JY (2000). A recombinant *Eimeria* protein inducing interferon-gamma production: comparison of different gene expression systems and immunization strategies for vaccination against coccidiosis. Avian Dis.

[58] Sander VA, Corigliano MG, Clemente M (2019). Promising plant-derived adjuvants in the development of coccidial vaccines. Front Vet Sci.

[59] Sun HX, Xie Y, Ye YP (2009). Advances in saponin-based adjuvants. Vaccine.

[60] Ma C, Li G, Chen W, Jia Z, Yang X, Pan X (2021). *Eimeria tenella*: IMP1 protein delivered by *Lactococcus lactis* induces immune responses against homologous challenge in chickens. Vet Parasitol.

[61] Hoan TD, Thao DT, Gadahi JA, Song X, Xu L, Yan R (2014). Analysis of humoral immune response and cytokines in chickens vaccinated with *Eimeria brunetti* apical membrane antigen-1 (EbAMA1) DNA vaccine. Exp Parasitol.

[62] Wallach M (2010). Role of antibody in immunity and control of chicken coccidiosis. Trends Parasitol.

[63] Lee SH, Lillehoj HS, Park DW, Jang SI, Morales A, García D (2009). Induction of passive immunity in broiler chickens against *Eimeria acervulina* by hyperimmune egg yolk immunoglobulin Y. Poult Sci.

[64] Lee SH, Lillehoj HS, Park DW, Jang SI, Morales A, García D (2009). Protective effect of hyperimmune egg yolk IgY antibodies against *Eimeria tenella* and *Eimeria maxima* infections. Vet Parasitol.

[65] Smetana H (1933). Coccidiosis of the liver of rabbits: experimental study of the mode of infection of the liver by sporozoites. Arch Pathol.

[66] Horton RJ (1967). The route of migration of *Eimeria stiedae* (Lindemann, 1865) sporozoites between the duodenum and bile ducts of the rabbit. Parasitology.

[67] Owen D (1970). Life cycle of *Eimeria stiedae*. Nature.

[68] Xun S, Fanyao K, Anxing Li (1994). Migration of the sporozoies of *Eimeria stiedai*. Acta Veterinaria et Zootechnica Sinica.

[69] Jiang L, Lin J, Han H (2012). Identification and characterization of *Eimeria tenella* apical membrane antigen-1 (AMA1). PLoS ONE.

[70] Jenkins MC, Fetterer R, Miska K (2015). Characterization of the *Eimeria maxima* sporozoite surface protein IMP1. Vet Parasitol.

